# An Afflicted Man

**Published:** 1879-05

**Authors:** 


An Afflicted Man.—A few days ago, a flying bookseller and stationer called at a publican's house in the village of——, and offered the landlord to sell him a copy of the "Afflicted Man's Companion." The landlord looked at it, and returned the copy, saying that he had a much better one already. The bookseller said he should like to see it. Come this way, said the publican, and, introducing him into a small parlor, showed the bookseller his dear wife lying on the floor dead drunk, with an empty whisky bottle by her side. There, said the publican, is my copy of the "Afflicted Man's Companion."

